# CpGene: a web application for epigenetic signature identification from DNA methylation arrays

**DOI:** 10.1093/bioinformatics/btag141

**Published:** 2026-03-25

**Authors:** Konstantinos Lazaros, Souzana Logotheti, Christopher Logothetis, Vasiliki Tzelepi, Panagiotis Vlamos, Aristidis G Vrahatis

**Affiliations:** Bioinformatics and Human Electrophysiology Laboratory, Department of Informatics, Ionian University, Corfu 49100, Greece; Department of Pathology, School of Medicine, University of Patras, Patras 26504, Greece; Department of Pathology, School of Medicine, University of Patras, Patras 26504, Greece; Department of Genitourinary Medical Oncology, The University of Texas MD Anderson Cancer Center, Houston, Texas 77030, United States; Department of Pathology, School of Medicine, University of Patras, Patras 26504, Greece; Bioinformatics and Human Electrophysiology Laboratory, Department of Informatics, Ionian University, Corfu 49100, Greece; Bioinformatics and Human Electrophysiology Laboratory, Department of Informatics, Ionian University, Corfu 49100, Greece

## Abstract

**Motivation:**

DNA methylation (DNAme) is the best studied epigenetic mechanism that plays pivotal role in tissue differentiation and epigenetic disruption has been correlated to diverse disease types (e.g. cancer, metabolic disorders). While various DNAme array platforms have been discovered, data analysis remains a challenging task which often requires in-depth bioinformatic expertise. Here, we developed a user-friendly web-based application for data analysis and visualization that accommodates users ranging from early-career basic/translational researchers to experienced bioinformaticians.

**Results:**

CpGene is a web application for analyzing DNA methylation array data. It supports Illumina 450K, EPIC, and EPICv2 methylation array platforms and processes .idat files with integrated preprocessing, normalization, and quality control. Biomarker discovery is available through either classic differential methylation point analysis or machine learning-based feature selection as well as gene enrichment analysis. Results are summarized with clear visualizations, to aid interpretation. By combining these functions in a unified interface, CpGene streamlines methylation analysis and helps identify CpG sites and genes with biological and clinical relevance.

**Availability and implementation:**

CpGene is openly accessible as a web service through http://cpgene.duckdns.org:8001/ and it’s source code is available on https://github.com/kostaslazaros/cpgenene.

## 1 Introduction

DNA methylation (DNAme) at CpG dinucleotides is an important epigenetic mechanism that regulates gene expression without altering the DNA sequence. It undergoes dynamic changes during development and differentiation, guiding cell identity and, when disrupted DNAme accounts for development and progression of various disease types (Chen et al. 2014, Hop et al. 2022, Logotheti et al. 2023). Beyond this, DNA methylation influences immune function, brain development, and cardiovascular health, with aberrant patterns linked to autoimmune diseases, neurological conditions, and heart disease (Fodder et al. 2023, Desiderio et al. 2024). Altered DNA methylation is also a hallmark of cancercarcinogenesis and cancer progression, where interplays with genomic instability in numerous haematological and solid tumors biomarkers (Chen et al. 2014, Papanicolau-Sengos and Aldape 2022).

DNA methylation arrays are widely used to study DNAme across the genome, providing important insights into epigenetic regulation of gene expression and cell behavior. Illumina platforms remain the gold standard, with the 450K array covering about 450 000 CpG sites, the EPIC array expanding this to 850 000, and the latest EPICv2 surpassing 900 000 by adding probes for key cis-regulatory regions such as enhancers and CTCF binding sites (Morris and Beck 2015, Mansell et al. 2019, Noguera-Castells et al. 2023). These arrays rely on conversion of methylated cytosines, which distinguishes them from unmethylated cytosines, enabling precise detection of methylation patterns that often extend across neighboring CpG sites in coordinated “in-phase” regions (Wang et al. 2019).

Although DNA methylation arrays are powerful tools for profiling epigenetic modifications at a large scale, they also present important limitations. From a computational standpoint, datasets derived from such arrays, typically contain significantly more features than samples, a problem known as the Hughes phenomenon or the curse of dimensionality (Mora 2023). This imbalance not only increases the complexity of the analysis but can also lead to biased or misleading results if not carefully addressed (Clark-Boucher et al. 2023).

### 1.1 Related work

Several software packages have been developed to support preprocessing, visualization, and differential methylation analysis of DNA methylation array data. RnBeads (Müller et al. 2019) provides a modular and comprehensive framework for preprocessing, normalization, and DMP identification, supporting large-scale analyses across Illumina platforms. ChAMP (Tian et al. 2017) emphasizes flexibility and automation, offering integrated modules for normalization, batch correction, and DMP analysis accompanied by effective visualization tools. ENmix (Xu et al. 2021) focuses on noise reduction and background correction using advanced statistical modeling, which enhances the accuracy of methylation estimates and improves downstream analyses. SeSAMe (Zhou et al. 2018) extends functionality to include preprocessing, DMP analysis, and probe-level bias correction, addressing signal artifacts and improving the reliability of differential methylation detection. WateRmelon (Pidsley et al. 2013) offers a streamlined framework for data normalization and quality assessment, providing multiple preprocessing options suited for Illumina arrays. MethyLumi (Davis and Bilke 2010) represents one of the earliest tools for Illumina methylation data, facilitating data import, quality control, and basic analytical tasks.

Although these tools have substantially advanced the computational analysis of DNA methylation, they are primarily implemented as R packages and require programming proficiency. None of them provide a fully integrated, web-based environment for end-to-end analysis, which limits accessibility for researchers without computational experience. In addition, current frameworks lack artificial intelligence-based CpG ranking algorithms, which can be particularly valuable in addressing the growing dimensionality and complexity of DNA methylation datasets. Integrating such methods can facilitate feature prioritization and enhance the identification of biologically meaningful biomarkers.

### 1.2 The CpGene application

Here, we introduce a web application named CpGene, that provides a user-friendly interface to facilitate the analysis of DNA methylation array data. The tool accommodates Illumina 450K, EPIC, and EPICv2 platforms, handling .idat files with built-in methods for preprocessing, normalization, and quality control. It provides options for biomarker discovery through either differential methylation analysis or machine learning-driven feature ranking, while functional interpretation is also supported through the use of EnrichR’s API. Results are delivered through intuitive visualizations that enhance interpretability. By combining these capabilities within a single environment, CpGene offers a practical solution for uncovering CpG sites and genes of biological and clinical importance. Ultimately, the CpGene platform is designed to be accessible and beneficial to researchers at all levels, from early-stage investigators such as undergraduate and master’s students, as well as PhD candidates, to senior researchers across a variety of scientific fields—including basic and translational scientists, clinicians, and experienced bioinformaticians. It offers a fast and reliable tool for epigenetic biomarker identification.

## 2 Materials and methods

### 2.1 Preprocessing and quality control

Preprocessing and quality control in CpGene are performed using the minfi R package (Aryee et al. 2014). Input consists of .idat files and a sample sheet, which are assembled into a single object for analysis. Low-quality probes are filtered out using detection *P*-values (>.01; the default for minfi), which identify signals that cannot be reliably distinguished from background fluorescence. Background noise correction is carried out with NOOB (Triche et al. 2013), followed by normalization steps that use principal component analysis of control probes to adjust for technical variation between samples, known as batch effects. Cross-reactive probes are then removed, and beta values are calculated to represent methylation levels at each CpG site. Finally, CpG sites of hemimethylated beta values (0.31–0.59), which cannot be clearly characterized as hypo- or hypermethylated, are excluded to refine the dataset (Nissenbaum et al. 2013). The outcomes of preprocessing are visualized with a bar plot of detection *P*-values, a dot plot for bisulfite conversion efficiency across red and green channels, a scatter plot for sample signal quality, and a beta value distribution plot after normalization. Users are also able to download the resulting processed files and all QC figures in high-resolution 300 dpi PNG format.

### 2.2 AI-based CpG ranking

Feature ranking is implemented through AI-based methods built on the scikit-learn Python library (Nelli 2023), offering flexibility depending on the strategy chosen by the user. The application incorporates wrapper-based approaches, embedded techniques, model-agnostic explainability methods, and neural-network-based strategies, ensuring that ranking can be performed under different modeling assumptions. Users may apply these methods either in a binary classification setting, comparing two conditions, or in a multi-class setting, involving more than two conditions. In addition, the interface allows users to interactively select the number of top-ranked features of preference prior to further investigation. The results are visualized through 2D PCA plots, where samples are shown as dots colored according to their target variable, with one plot generated before feature ranking and another after, using only the top k features specified by the user.

### 2.3 Differential methylation analysis

**Figure 1:**
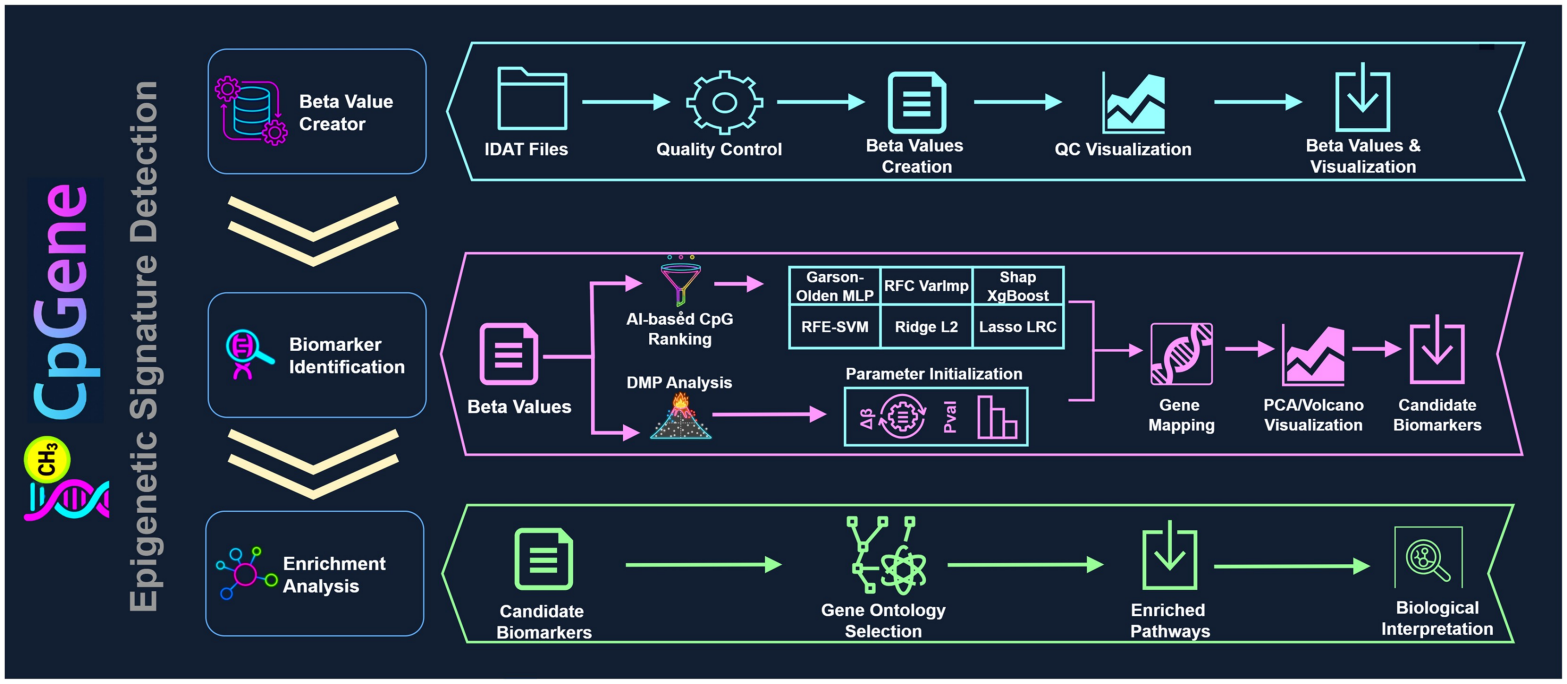
Overview of the CpGene workflow. The pipeline consists of three main stages: Beta Value Creator, Biomarker Identification, and Enrichment Analysis. Starting from Illumina IDAT files, CpGene performs preprocessing and quality control to generate normalized beta values and corresponding QC visualizations. These values are used for biomarker discovery through either machine learning-based feature selection or differential methylation point (DMP) analysis. During feature selection, sample distributions are visualized via principal component analysis (PCA) plots generated both before and after the selection process, whereas in DMP analysis, methylation differences across CpG sites are illustrated using volcano plots. Identified CpG sites are subsequently mapped to their corresponding genes. The final stage conducts pathway and term enrichment through Enrichr-based functional annotation, facilitating biological interpretation and the identification of enriched molecular pathways of potential clinical relevance.

Differentially methylated position (DMP) analysis is supported through integration with the limma R package (Budkina et al. 2023), enabling comparisons between two conditions to identify CpG sites with significant methylation differences. Users can adjust delta beta and *P*-value thresholds directly through the interface to control stringency, and they may further select the number of top features to retain, ensuring that downstream analyses focus on the most relevant signals. The results are visualized with a volcano plot, where hypermethylated CpG sites are highlighted in red, hypomethylated sites in green, and non-significant sites in gray, providing a clear overview of differential methylation patterns (supplementary material). Fig. 1 provides a graphical abstract with the core utilities of the application.

### 2.4 Gene enrichment analysis

CpG sites selected through either feature ranking or differential methylation analysis are mapped to their corresponding gene symbols based on the array type, using the appropriate manifest file. Gene set enrichment analysis is then carried out through the Enrichr API (Xie et al. 2021), which provides access to a wide range of molecular pathway databases. The results are visualized through the use of a horizontal bar plot which can be sorted either by *P*-value or by combined enrichment score.

## 3 Conclusion

CpGene is a practical, efficient, and accessible tool for the analysis of DNA methylation array data. Table 1 offers a concise comparison of CpGene’s features with other well-established tools, highlighting its distinctive strengths. By integrating preprocessing, normalization, quality control, feature ranking, differential methylation analysis, and gene set enrichment within a single interface, it provides a systematic environment for uncovering biologically and clinically relevant CpG sites and genes. The interactive design and multiple analysis strategies render CpGene a versatile application that supports robust biomarker discovery and facilitates the exploration of disease-associated epigenetic alterations. In addition, the platform’s comprehensive visualization capabilities—ranging from bar plots and scatter plots to volcano plots and enrichment charts—not only aid in the interpretation of complex genomic data but also enhance user engagement and understanding. This comprehensive approach streamlines epigenetic studies by minimizing technical barriers and maximizing analytic flexibility, positioning CpGene as a valuable resource for the scientific community in the ongoing pursuit of actionable epigenetic biomarker identification and insights into disease mechanisms.

**Table 1:** Comparison of CpGene with existing DNA methylation array analysis tools.^a^^,^^b^

	CpGene	RnBeads	ChAMP	SeSAMe	ENmix	wateRmelon	MethyLumi
450K	√	√	√	√	√	√	√
EPIC	√	√	√	√	√	√	×
EPICv2	√	√	×	√	√	×	×
Preprocessing/QC	√	√	√	√	√	√	√
AI-based CpG ranking	√	×	×	×	×	×	×
Differential methylation analysis	√	√	√	√	√	√	×
Gene enrichment analysis	√	√	√	×	×	×	×
Web application	√	×	×	×	×	×	×

a CpGene integrates support for all Illumina array generations (450K, EPIC, EPICv2), preprocessing and quality control, differential methylation analysis, AI-based CpG ranking for biomarker discovery, and gene enrichment analysis within a user-friendly web application. In contrast, existing tools offer subsets of these functionalities, with limited or no support for advanced AI methods and web-based accessibility.

b √ indicates support/availability; × indicates not supported.

## Author contribution

Konstantinos Lazaros (Conceptualization [equal], Methodology [equal], Software [lead], Visualization [equal], Writing—original draft [equal], Writing—review & editing [equal]), Souzana Logotheti (Conceptualization [equal], Methodology [equal], Validation [lead], Visualization [equal], Writing—original draft [equal], Writing—review & editing [equal]), Christopher Logothetis (Conceptualization [equal], Validation [equal], Writing—original draft [equal], Writing—review & editing [equal]), Vasiliki Tzelepi (Conceptualization [equal], Funding acquisition [lead], Validation [equal], Writing—original draft [equal], Writing—review & editing [equal]), Panagiotis Vlamos (Conceptualization [equal], Validation [equal], Writing—original draft [equal], Writing—review & editing [equal]), and Aristidis G. Vrahatis (Conceptualization [equal], Methodology [equal], Software [lead], Visualization [equal], Writing—original draft [equal], Writing—review & editing [equal])

## Supplementary material

Supplementary material is available at *Bioinformatics* online.

## Supplementary Material

btag141_Supplementary_Data

## Data Availability

There are no new data associated with this article.
