# Supplementary material for: CpGene: a web application for epigenetic signature identification from DNA methylation arrays
Source: Bioinformatics. 2026 Mar 25;42(5):btag141. doi: 10.1093/bioinformatics/btag141 (PMC13141144; doi:10.1093/bioinformatics/btag141)
Supplement: btag141_Supplementary_Data [file btag141_supplementary_data.pdf]

Supplementary Material

CpGene: A Web Application for Epigenetic Signature Identification from DNA Methylation Arrays

Konstantinos Lazaros[1,2*], Souzana Logotheti[2,3] Christopher Logothetis[3]

Vasiliki Tzelepi[2] Panagiotis Vlamos[1] and Aristidis G. Vrahatis[1]

[1]Bioinformatics and Human Electrophysiology Laboratory, Department of Informatics, Ionian University, 49100, Corfu, Greece, [2]Department of Pathology, School of Medicine, University of Patras, 26504, Patras, Greece and [3]Department of Genitourinary Medical Oncology, The University of Texas MD Anderson Cancer Center, 77030, Texas, USA

∗Corresponding author. lakonstant@ionio.gr

Application Architecture Overview

System Architecture

CpGene is a DNA Methylation Analysis Platform built with a modern microservices architecture using FastAPI, Celery, Redis, and Docker. The system processes IDAT files (Illumina methylation arrays) and CSV data to perform comprehensive feature selection and machine learning analysis.

Architecture Components

1. Frontend Layer

- **Technology**: HTML5, JavaScript, Tailwind CSS
- **Main Files**:
  - `templates/index.html` - Landing page
  - `templates/bval.html` - Primary interface for IDAT file upload
  - templates/dmp.html - DMP Analysis
  - templates/enrich.html - Enrichment Analysis
  - templates/feature-selection.html - - Feature Selection
  - templates/genesel.html - Biomarker Identification
  - `static/bval.js` - Interactive functionality, image galleries, download management
  - `static/*.css` - Styling and responsive design

Features:

- Drag-and-drop file upload with SHA-1 hashing
- Real-time image gallery with click-to-enlarge modals
- Responsive design with emerald neon styling
- ZIP download functionality for analysis results

2. API Layer (FastAPI)

- **Framework**: FastAPI with Uvicorn ASGI server
- **Port**: 8001
- **Main File**: `app/start_fastapi.py`

Router Modules:

- `routers/html.py` - Template rendering endpoints
- `routers/file_upload.py` - File upload and management
- `routers/prognosis_analysis.py` - Analysis endpoints, image serving, ZIP downloads
- `routers/util.py` - Utility endpoints

Key Endpoints:

- `POST /idat/upload` - IDAT file bundle upload
- `GET /idat/exists/{id}` - Check if bundle exists
- `GET /prognosis/images/{sha1_hash}` - List generated PNG images
- `GET /prognosis/image/{sha1_hash}/{filename}` - Serve individual images
- `GET /prognosis/download-all/{sha1_hash}` - Download ZIP of all results

3. Task Processing Layer (Celery-Redis)

- **Message Broker**: Redis
- **Task Modules**:
  - `celery_tasks/file_tasks.py` - File processing operations
  - `celery_tasks/prognosis_tasks.py` - ML analysis tasks
  - `celery_tasks/celery.py` - Celery configuration

Task Processing Features:

- Asynchronous IDAT file processing
- CSV data analysis and feature selection
- PNG visualization generation
- JSON serialization handling for pandas DataFrames

4. Machine Learning Engine

- **Location**: `app/algorithms/`

Feature Selection Methods:

- **Random Forest Variable Importance** (`random_forest_varimp.py`) - Tree-based feature ranking
- **LASSO Logistic Regression** (`lasso_logistic_regression.py`) - L1 regularization
- **RFE SVM** (`rfe_svm.py`) - Recursive Feature Elimination with Support Vector Machines
- **Ridge L2** (`ridge_l2.py`) - L2 regularized linear models
- **SHAP XGBoost** (`shap_xgboost.py`) - Explainable AI feature importance
- **Garsen-Olden MLP** (`garsen_olden_mlp.py`) - Neural network with connection weights analysis

5. Containerized Processing (Docker)

- **R-MinFi Container**: Specialized container for IDAT file processing
- **Purpose**: Methylation array data preprocessing
- **Operations**:
  - Beta value calculation
  - Quality control metrics
  - Methylation signal extraction
  - Array type detection
- **R-DMP (limma) Container**: Specialized container for DMP analysis
- **Purpose**: Differentially Methylated Positions (DMP) analysis from methylation arrays using limma (supports covariates & contrasts).
- **Operations**:
  - β-value matrix ingestion with matched phenotype table
  - Category/contrast selection (e.g., A vs B or custom contrasts)
  - limma linear modeling with empirical Bayes
  - P-value and FDR (BH) computation
  - CpG filtering by |Δβ| and p/FDR thresholds

6. Data Storage

- **File Storage**: Local filesystem (`uploads/` directory)
- **Structure**: `uploads/{sha1_hash}/in/` and `uploads/{sha1_hash}/out/`
- **Input Files**: `.idat`, `.csv`
- **Output Files**: PNG visualizations, `array_type.csv`, `bval_data.csv`

7. Infrastructure Components

Redis (Message Broker)

- **Port**: 6379
- **Purpose**: Celery task queue and results backend
- **Docker**: Managed via `docker/docker-redis-commander/`

Package Management

- **Python**: uv (modern Python package manager)
- **Frontend**: `npm` for Tailwind CSS and build tools
- **Configuration**: `pyproject.toml`, `package.json`

Data Flow

1. File Upload Process

```
User Browser → FastAPI → SHA-1 Calculation → File Storage → Celery Task Queue
```

2. Analysis Pipeline

```
Celery Worker → Docker R-MinFi → ML Algorithms → PNG Generation → Result Stora
ge
```

3. Result Delivery

```
FastAPI Image Endpoints → Dynamic Gallery → User Download (Individual/ZIP)
```

Security & Performance Features

File Integrity

- **SHA-1 Hashing**: Ensures file integrity and prevents duplicate processing
- **Bundle Detection**: Automatic detection of existing analyses

Scalability

- **Async Processing**: Non-blocking task execution
- **Worker Scaling**: Multiple Celery workers can be deployed
- **Resource Management**: Configurable task timeouts and memory limits

User Experience

- **Real-time Feedback**: Progress indicators and status updates
- **Responsive Design**: Mobile-friendly interface
- **Batch Operations**: ZIP download of all analysis results

Development Workflow

Starting the System

```
# 1. Start Redis
cd docker/docker-redis-commander && docker compose up -d

# 2. Start Celery Worker
uv run celery -A app.celery_tasks worker -l INFO

# 3. Start FastAPI Server
uv run uvicorn app.start_fastapi:app --host 0.0.0.0 --port 8001 --reload
```

Development Tools

- **Auto-reload**: FastAPI development server with hot reloading
- **Task Monitoring**: Redis Commander for queue inspection
- **API Documentation**: Automatic OpenAPI docs at `/docs`

Integration Points

External Dependencies

- **Scientific Libraries**: pandas, numpy, scikit-learn, xgboost
- **Web Framework**: FastAPI, Jinja2 templates
- **Task Queue**: Celery with Redis backend
- **Containerization**: Docker for R environment isolation

Data Formats

- **Input**: Illumina IDAT files, CSV methylation data
- **Processing**: Pandas DataFrames, NumPy arrays
- **Output**: PNG visualizations, CSV results, ZIP archives

This architecture provides a robust, scalable platform for DNA methylation analysis with a modern web interface and powerful machine learning capabilities.

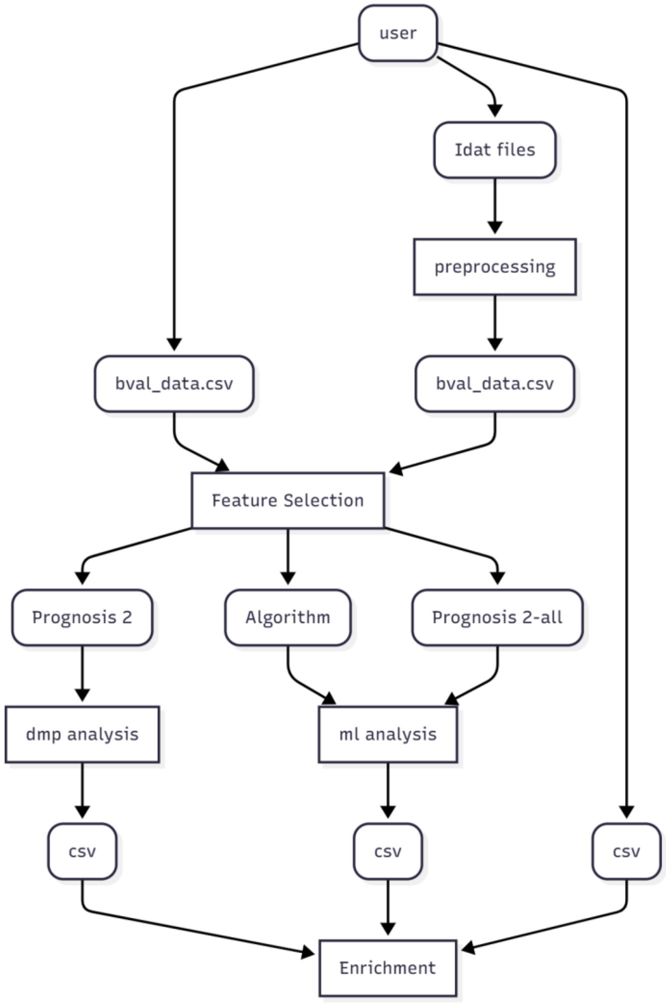

Supplementary Figure 1. User-provided IDAT files undergo preprocessing and quality control to produce normalized beta values, which are exported as processed datasets. These data are then subjected to feature selection using either differential methylation point analysis or machine learning–based ranking in binary or multi-class settings. Each analytical path generates CpG sets that are saved in CSV format. The resulting features are mapped to their corresponding genes and subsequently analyzed through enrichment procedures to derive biologically meaningful interpretations of the identified epigenetic signatures.

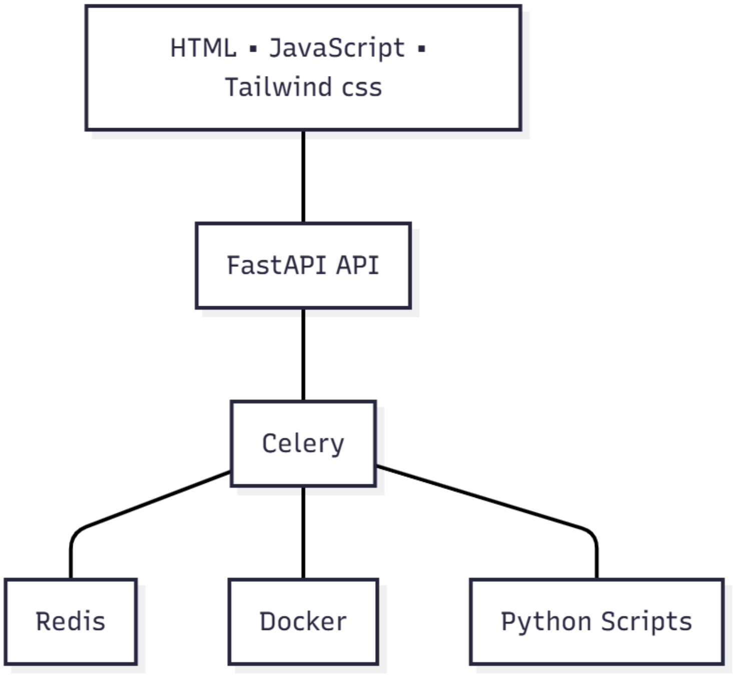

Supplementary Figure 2. The frontend layer, built with HTML, JavaScript, and Tailwind CSS, communicates with the FastAPI backend, which coordinates all user requests. Task scheduling and asynchronous job management are handled through Celery, enabling efficient execution of long-running analyses. Celery interacts with Redis for message brokering, executes containerized workflows through Docker, and triggers the required Python scripts, forming a scalable and modular infrastructure for high-throughput DNA methylation analysis.

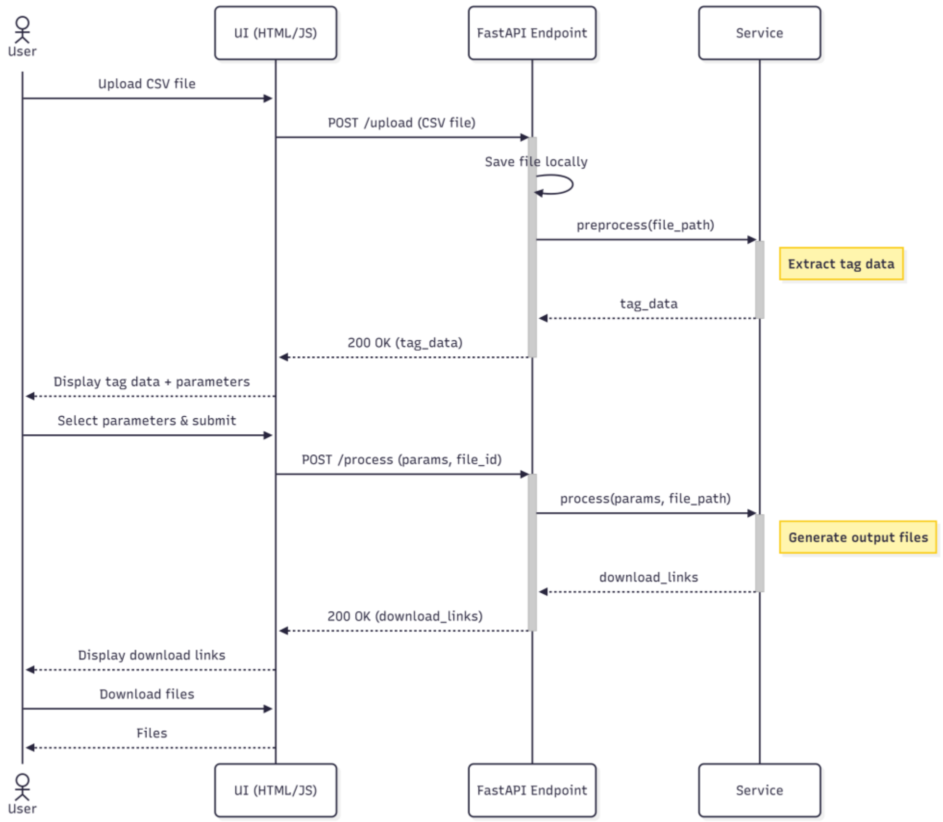

Supplementary Figure 3. The diagram outlines the communication flow between the user interface, the FastAPI backend, and the underlying service layer. The user initiates the process by uploading a CSV file through the web interface, which is transmitted to the FastAPI endpoint and stored locally. The backend then invokes preprocessing functions to extract tag information, which is returned to the interface for parameter selection. After the user submits the selected parameters, the backend triggers the processing module that generates the required output files. The resulting download links are sent back to the interface, enabling the user to retrieve the final processed data. This sequence demonstrates the coordinated exchange of data and tasks across the system's components.

Preprocessing & Quality Control

Preprocessing of Illumina methylation arrays in CpGene follows a structured and quality-controlled workflow based on the minfi framework, ensuring high data integrity and reproducibility before any downstream analysis. Raw IDAT files are imported alongside the sample sheet, which provides metadata and identifiers linking each array to its biological condition. Quality control begins with the computation of probe-level detection *p*-values, which quantify the confidence that measured intensities differ from background noise. The mean detection *p*-value across all probes is calculated for each sample, serving as an indicator of overall assay quality. Samples exceeding the established threshold are excluded, thereby eliminating low-confidence data that could bias subsequent analyses. Assay performance is further assessed using

control probes designed to monitor bisulfite conversion efficiency across both color channels, as well as by examining global quality metrics that summarize intensity distributions per sample.

Once poor-quality samples are removed, normalization is applied through a control-probe–informed approach that corrects for between-array technical variability while preserving biological differences. This method begins with a background correction step that models non-specific fluorescence using a combined normal–exponential framework. Here, background noise is assumed to follow a normal distribution, while the true methylation signal is modeled as exponentially distributed. Out-of-bound probe data—fluorescence measured from the inactive color channel of Type I probes—provide an empirical estimate of background levels for each sample. By subtracting this modeled background, the resulting intensities more accurately represent the true methylation signal.

Following background correction, the workflow applies principal component analysis (PCA) to the control probe intensities to capture dominant sources of unwanted technical variation, such as batch or hybridization effects. The first principal components are then used to adjust probe intensities, effectively removing systematic technical noise while maintaining biological signal integrity. This combined background correction and PCA-based normalization strategy has demonstrated high performance in studies involving strong biological contrasts, such as comparisons between cancer and normal tissues or heterogeneous cell types. It ensures that the resulting β-values are technically consistent and biologically meaningful.

After normalization, several filtering steps are applied to retain only high-confidence CpG probes. Probes that fail the detection *p*-value threshold in any remaining sample are removed to eliminate unreliable measurements. Loci overlapping known single-nucleotide polymorphisms are excluded to prevent genotype-driven artifacts. Additionally, published catalogs of cross-reactive probes (those that hybridize to multiple genomic locations) are used to further refine the dataset. The remaining β-values are then examined to confirm their expected bimodal distribution, reflecting hypomethylated and hypermethylated states across samples. CpG sites that fall consistently within the intermediate hemi-methylated range (β-values between 0.3 and 0.6) are excluded, as they typically represent ambiguous methylation states and contribute limited discriminatory power to downstream analyses (Nissenbaum et al, 2013).

This filtering step is motivated by the observation that probes with persistently intermediate beta values tend to offer limited discriminatory value in comparative studies, often reflecting constitutively bivalent chromatin regions, imprinted loci, or heterogeneous cell-type mixtures rather than condition-specific methylation changes. It is nonetheless recognized that intermediate methylation can be biologically relevant in certain contexts, such as studies of allele-specific methylation, cell differentiation, or tissue heterogeneity. For this reason, the filtering step can be disabled via a toggle in the preprocessing interface, allowing users to retain hemi-methylated sites when their study design requires it.

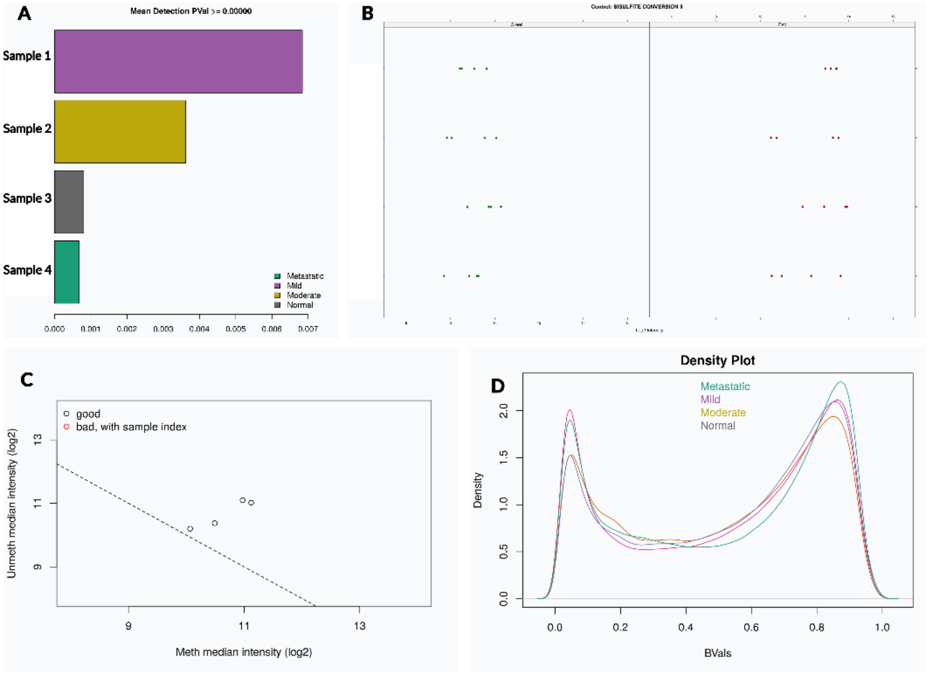

Supplementary Figure 4. (A) Mean detection *p*-values per sample, with color-coded categories indicating clinical groups. Samples exceeding the detection threshold were excluded from further analysis. (B) Control probe performance for bisulfite conversion type II, shown across both red and green channels, confirming consistent conversion efficiency. (C) Median methylated versus unmethylated signal intensities used for identifying low-quality samples based on overall signal distribution. (D) Density plot of β-values across all samples and groups following normalization and filtering, illustrating the expected bimodal distribution corresponding to hypo- and hypermethylated CpG sites.

AI-based CpG Ranking

The growing complexity of high-throughput biological data, especially from DNA methylation arrays, has created the need for more advanced computational methods in biomarker discovery. Modern array platforms measure hundreds of thousands of CpG sites, creating a large imbalance between the number of features and the number of samples, often referred to as the curse of dimensionality. This imbalance can make it difficult for traditional statistical techniques to identify meaningful patterns. To address these challenges, CpGene uses a set of artificial intelligence–based feature selection methods, including the Garson Olden MLP, Ridge Classifier with L2 regularization, Recursive Feature Elimination (RFE) with an SVM classifier, SHAP with XGBoost, Random Forest Variable Importance, and Logistic Regression with Lasso. In all cases, feature ranking is run on the full dataset.

Garson-Olden MLP

Feature selection using the Garson–Olden Multilayer Perceptron (MLP) method in CpGene quantifies the relative contribution of each CpG site by propagating connection weights through the trained network. Once the MLP model is fitted, each neuron's influence on the output is

expressed through the absolute magnitudes of its connection weights. For a network with one hidden layer, the importance I_i of an input feature i can be computed as:

$$\boldsymbol{I_i} = \frac{\sum_{h=1}^{H} |\boldsymbol{w_{ih}} \times \boldsymbol{u_h}|}{\sum_{j=1}^{N} \sum_{h=1}^{H} |\boldsymbol{w_{jh}} \times \boldsymbol{u_h}|}$$

where w_{ih} denotes the weight connecting input neuron i to hidden neuron h, v_h represents the weight connecting hidden neuron h to the output layer, H is the number of hidden neurons, and N the total number of inputs. This expression captures the cumulative strength of all weighted paths linking each input to the output, thereby reflecting its relative influence on the prediction. In deeper networks, this principle is extended by recursively multiplying the absolute values of weight matrices across layers, yielding a generalizable metric of importance that can handle non-linear feature interactions (Garson, 1991; Olden et al., 2004).

To ensure stable and reproducible feature rankings, the model is trained repeatedly using different random seeds, and the resulting importance vectors are averaged. This reduces the effect of stochastic variations inherent to neural network initialization and training. The final ranked list of CpG sites reflects features that exert the most consistent influence across trained models, providing interpretable and biologically relevant markers for subsequent mapping and enrichment analyses.

RFE-SVM

Feature ranking using Recursive Feature Elimination with a Support Vector Machine (RFE-SVM) (Azman et al., 2023) is based on iteratively training a linear classifier to quantify the relative contribution of each CpG site to the discrimination between biological conditions. In this framework, a linear SVM learns a decision boundary defined by a hyperplane that maximizes the margin between classes. Each CpG site is assigned a coefficient w_j, representing its contribution to the separating hyperplane. The absolute magnitude | w_j | serves as an indicator of importance, as features with larger coefficients exert greater influence on the classification. At each iteration t, the feature importance scores are computed as

$$\boldsymbol{s_j^{(t)}} = \left|\boldsymbol{w_j^{(t)}}\right|$$

where $w_j^{(t)}$corresponds to the weight of feature $j$at iteration t. The features with the smallest $s_j^{(t)}$values are then removed, and the classifier is retrained on the remaining subset. Repeating this process yields a complete ranking of features from most to least informative, allowing the identification of CpG sites that contribute most consistently to class separation. This methodology offers both interpretability and robustness, as the linear SVM provides a direct link between model coefficients and biological variables. The elimination process progressively refines the feature space by removing redundant or weakly contributing CpG sites, ultimately producing a ranking that reflects their discriminative strength. Mathematically, the decision function of the SVM is expressed as

$$\boldsymbol{f(x)} = \boldsymbol{w^T x + b}$$

where $\mathbf{w} = [w_1, w_2, \dots, w_p]$ denotes the weight vector and $b$the bias term. The recursive nature of RFE ensures that only features maintaining high | w_j | values across successive iterations are

retained near the top of the ranking, capturing the most biologically relevant methylation markers for further analysis and interpretation.

Random Forest Variable Importance

Feature ranking with Random Forest variable importance (Huang and Chen, 2021) relies on how much each CpG site contributes to improving class separation across an ensemble of decision trees. During training, each tree partitions the samples by selecting splits that reduce node impurity (e.g., Gini impurity). The importance of feature $j$in a given tree $t$is computed as the sum of impurity decreases produced by all splits on that feature, each weighted by the fraction of samples reaching the split:

$$\boldsymbol{Imp_j^{(t)}} = \sum_{s \epsilon S_j^{(t)}} \boldsymbol{p(s)\Delta_i(s)}, \textbf{ with } \boldsymbol{\Delta_i(s) = i(parent) - i(left) - i(right)}$$

where $\mathcal{S}_j^{(t)}$is the set of splits on feature $j$in tree t, $p(s)$is the proportion of training samples at the split, and $i(\cdot)$is the node impurity (for Gini, $i(n) = 1 - \sum_k p_k(n)^2$, with $p_k(n)$ the class proportion at node n). The Random Forest importance is then obtained by averaging over trees and normalizing so that $\sum_j \mathrm{Imp}_j = 1$. This yields a direct, model-based measure of how strongly each CpG site contributes to reducing classification uncertainty across the ensemble.

In practice, the procedure produces a complete ordering of CpG sites by descending importance. Aggregation over many trees improves stability in high-dimensional settings and reduces sensitivity to individual partitions. It should be noted that impurity-based importance can distribute credit among correlated features and may favor variables with many possible split points; these effects are typical of tree-based models and can be contextualized alongside domain knowledge or complementary ranking methods if needed. The resulting ranked list highlights CpG sites that consistently drive impurity reductions across the forest, providing an interpretable basis for downstream gene mapping and pathway analysis.

Ridge-L2

Feature ranking with Ridge (L2-regularized) classification (Khan et al., 2019) uses the magnitude of the model coefficients as an index of each CpG site's contribution to class discrimination, while the L2 penalty stabilizes estimates in the high-dimensional setting. For a binary problem, Ridge learns a weight vector $\mathbf{w}$ by minimizing a penalized loss of the form

$$\min_{w,b} \mathcal{L}(y, w^T x + b) + \alpha \|w\|_2^2$$

where $\mathcal{L}$ is a convex classification loss and $\alpha > 0$controls shrinkage of coefficients toward zero. In the linear setting, larger absolute coefficients $| w_j |$ indicate features that exert stronger influence on the decision function $f(\mathbf{x}) = \mathbf{w}^\top \mathbf{x} + b$. For multiclass classification, the model yields one coefficient vector per class in a one-vs-rest formulation; feature importance can then be aggregated across classes by combining the class-specific absolute coefficients. To obtain a stable ranking that is less sensitive to sample fluctuations, the procedure repeats model fitting over multiple random subsamples of the rows and averages the resulting coefficient magnitudes. Denoting by $\mathbf{w}^{(r)}$ the coefficient vector estimated on repeat $r = 1, \ldots, R$, the stability-averaged importance for feature $j$in the binary case is

$$s_j = \frac{1}{R}\sum_{r=1}^{R}\left|w_j^{(r)}\right|$$

In the multiclass case with Kclasses and class-specific weights $w_{kj}^{(r)}$, the aggregated score is

$$s_j = \frac{1}{R}\sum_{r=1}^{R}\frac{1}{K}\sum_{k=1}^{K}\left|w_{kj}^{(r)}\right|$$

Sorting features by s_j from largest to smallest yields a complete ranking of CpG sites. The L2 penalty reduces variance and distributes weight among correlated predictors, while the repetition-and-averaging step enhances robustness, providing a practical and interpretable pathway to prioritize methylation markers for downstream analysis.

SHAP XgBoost

Feature ranking with SHAP values for gradient-boosted trees quantifies each CpG site's local contribution to model predictions. For a fitted XGBoost classifier (Zhang et al., 2023) with prediction function $f(\mathbf{x})$, SHAP assigns to every feature $j$and sample $i$an additive attribution ϕ_{ij} such that

$$f(x_i) = \varphi_{i0} + \sum_{j=1}^{p}\varphi_{ij}$$

where $\phi_{i0} = \mathbb{E}[f(\mathbf{X})]$ is the model's expected output. Each ϕ_{ij} is the Shapley value of feature $j$computed over all coalitions of features, capturing its marginal contribution to the prediction while preserving local accuracy and consistency. For tree ensembles, TreeExplainer computes ϕ_{ij}efficiently by aggregating path-dependent contributions across trees, avoiding brute-force enumeration of coalitions. In multiclass settings, attributions are defined per class; they can be arranged as $\Phi_i \in \mathbb{R}^{K\times p}$ and then combined across classes (e.g., by averaging absolute values). To obtain a global importance score for ranking CpG sites, per-sample attributions are summarized by the mean absolute SHAP value,

$$S_j = E_i[|\varphi_{ij}|]$$

or, in the multiclass case, $S_j = \mathbb{E}_i[\frac{1}{K}\sum_{k=1}^{K} \mid \phi_{ij}^{(k)} \mid]$. Sorting features by S_j yields a complete ordering from most to least influential on model output. When two features have similar S_j, a secondary, model-internal criterion such as XGBoost's split gain (the average loss reduction attributable to splits on a feature) can be used to break ties. The result is an interpretable, model-consistent ranking that reflects both local and global impact of CpG sites on predictions, suitable for downstream gene mapping and enrichment analysis.

Lasso Logistic Regression

Feature ranking with Lasso-regularized logistic regression treats each CpG site's coefficient as a direct measure of contribution to class discrimination, while the ℓ_1 penalty promotes sparsity

(Friedman et al., 2010). For a binary outcome, the model fits a linear decision function $f(\mathbf{x}) = \mathbf{w}^\top \mathbf{x} + b$and estimates $(\mathbf{w}, b)$by minimizing the penalized negative log-likelihood

$$\min_{w,b} \frac{1}{n} \sum_{i=1}^{n} \left[-y_i \log \sigma\big(f(x_i)\big) - (1 - y_i) \log(1 - \sigma(f(x_i))) \right] + \lambda \|w\|_1$$

where $\sigma(z) = 1/(1 + e^{-z})$ and $\lambda > 0$controls sparsity. The ℓ_1 term drives many coefficients exactly to zero, yielding an embedded selection of CpG sites that most strongly affect the log-odds of class membership. In the multiclass case with K classes, a one-vs-rest formulation produces class-specific weight vectors $\mathbf{w}_1, \ldots, \mathbf{w}_K$ with the same penalized likelihood principle applied per class.

Feature importance is then derived from the fitted coefficients. For binary classification, the absolute magnitude $| w_j |$serves as the importance of feature j; larger values indicate stronger influence on the decision function. For multiclass outcomes, a common aggregation is the coefficient vector norm across classes, e.g. $s_j = \| [w_{1j}, \ldots, w_{Kj}]^\top \|_2$, which summarizes how much feature $j$contributes across all one-vs-rest problems. Sorting features by these scores yields a complete ranking from most to least informative. Because $\ell_1$regularization can distribute weight unevenly among correlated CpGs, simple secondary criteria based on feature–response association (e.g., correlation with class indicators) can be applied to break ties between features with similar primary scores, leading to a stable and interpretable ordering for downstream annotation and enrichment analysis.

Default Hyperparameter Configurations

To ensure transparency and reproducibility, the default hyperparameter settings for each implemented feature selection method are described below. These values represent the configurations used when the application is run without user modification and serve as a reference for users wishing to replicate or adapt the analyses. The RFE-SVM uses a linear kernel with a regularization parameter of C = 1.0, and at each iteration a single feature is removed (step = 1). The use of a linear kernel means that the resulting feature weights have a direct geometric interpretation as contributions to the separating hyperplane, which makes the ranking straightforward to interpret. The Random Forest is built from 100 decision trees with no constraint on maximum depth, meaning each tree grows until its leaves are pure or contain a single sample. Feature importance is computed as the mean decrease in Gini impurity across all splits on a given feature, averaged over the full ensemble and normalized to sum to one. For SHAP XGBoost, the gradient-boosted model is trained with a maximum tree depth of 6, a learning rate of 0.3, a subsample ratio of 1.0, and 100 boosting rounds. SHAP values are computed using TreeExplainer, which derives exact Shapley values by propagating contributions through each tree path rather than relying on approximations. The global importance score for each CpG site is the mean of its absolute SHAP values across all samples.

Both the Lasso Logistic Regression and the Ridge L2 Classifier use regularization strengths selected through internal 5-fold cross-validation, employing LassoCV and RidgeCV respectively, each scanning a log-spaced grid of candidate values. The Lasso solver is set to 'saga', which handles L1 penalties efficiently in high-dimensional settings. For multiclass problems, a one-vs-rest strategy is applied in both cases. To further stabilize the Ridge rankings, the model is fitted repeatedly on

different random subsamples of the data and the absolute coefficient magnitudes are averaged across runs. The Garson-Olden MLP uses a single hidden layer of 100 neurons with ReLU activations, trained using the Adam optimizer for a maximum of 500 epochs. Early stopping is enabled with a validation fraction of 10% and a patience of 10 iterations, so training terminates once performance on the held-out portion stops improving. Because neural network training is sensitive to weight initialization, the model is run multiple times with different random seeds and the resulting importance vectors are averaged before producing the final ranking.

AI-based CpG Ranking Results Download & Visualization

In all implemented feature selection methods, users are provided with the option to specify the number of top-ranked CpG sites to retain for downstream analysis. This flexibility allows the selection of an appropriate subset of features based on the user's analytical goals or computational constraints. To facilitate the visual interpretation of CpG ranking results, dimensionality reduction is performed using Principal Component Analysis (PCA) (Greenacre et al., 2022). Two scatter plots are generated to illustrate the impact of feature selection on sample separation. The first plot is constructed using all available features, while the second utilizes only the top N features chosen by the user. In both visualizations, each point represents a sample, and colors correspond to the target variable or class label. When feature selection effectively captures the most discriminative CpG sites, the resulting reduced representation tends to display distinct and well-defined clusters for each category, highlighting the enhanced separability achieved after ranking and selection. This visualization is implemented through the use of the matplotlib and seaborn python libraries.

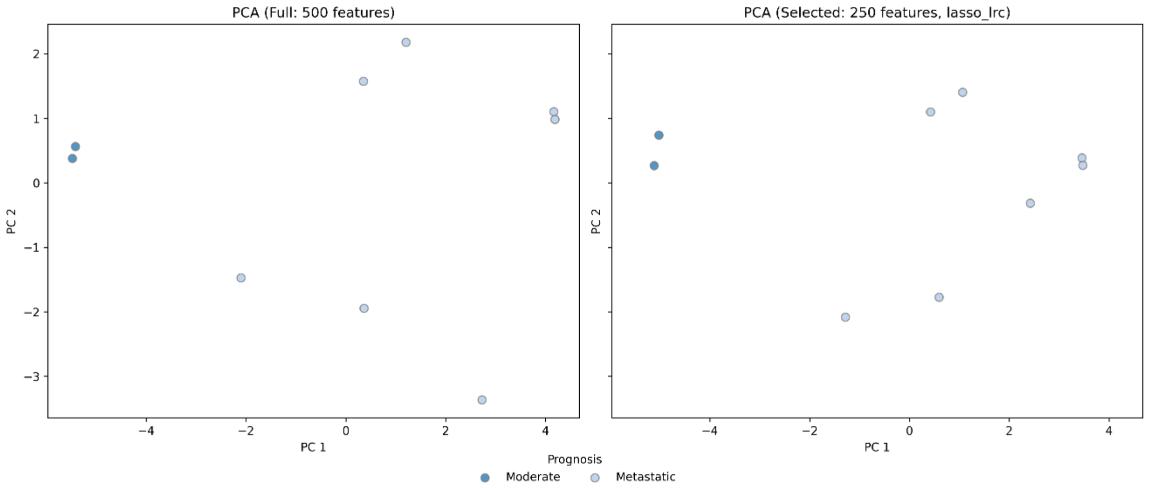

Supplementary Figure 5. Example of PCA visualization illustrating the effect of CpG feature selection. In this case, Lasso logistic regression was applied to a dataset containing two prognostic categories, *Moderate* and *Metastatic*. The left plot shows the PCA projection using all 500 CpG features, while the right plot displays the projection after retaining the top 250 ranked features.

Each point represents a sample, colored according to its prognostic category. After feature selection, the two groups become more distinguishable, demonstrating the enhanced separability achieved through the ranking process.

It should be noted that feature ranking in CpGene is performed on the full input dataset rather than within cross-validation folds. This is consistent with the intended use of the tool as a means of generating candidate CpG sets for downstream investigation, not as a predictive modelling framework. As a result, the PCA visualizations produced before and after feature selection reflect the discriminative structure of the data at hand and should not be interpreted as estimates of performance on unseen samples. For methods that involve stochastic elements — specifically the Garson-Olden MLP and the Ridge L2 classifier — the implementation runs the algorithm multiple times across different random seeds and averages the resulting importance scores, which improves the stability of the final ranking. Methods such as Lasso and RFE-SVM are deterministic and therefore produce the same ranking regardless of the run. Users are encouraged to treat the identified CpG sets as prioritized candidates requiring further validation, and to consider cross-validated predictive modelling as a separate, subsequent step once a candidate list has been established.

DMP Analysis

Differential methylation point (DMP) analysis is performed using the limma package (Adams et al., 2023), which applies linear modeling to identify CpG sites exhibiting significant methylation differences between two user-defined biological conditions. The user specifies which two categories of the target or output variable to compare, such as disease states, treatment groups, or prognostic outcomes. The analysis uses a matrix of methylation β-values, from which a design matrix is constructed to represent the selected conditions. For each CpG site, a linear model is fitted to estimate the difference in average methylation (Δβ or *deltaBeta*) between the two groups. A contrast matrix isolates the specific comparison of interest, and empirical Bayes moderation is applied to improve the precision of variance estimates and increase the reliability of differential detection, particularly when the number of samples is limited.

The resulting statistics include both Δβ values and *p*-values for every CpG site, reflecting the magnitude and significance of methylation changes, respectively. Both thresholds are defined by the user, allowing customization of the analysis according to the desired stringency level. CpG sites with positive Δβ values exceeding the chosen threshold are classified as hypermethylated in the first condition, whereas those with negative Δβ values below the threshold are considered hypomethylated. By enabling user control over the compared conditions and statistical cutoffs, this approach provides flexibility and adaptability across diverse methylation studies.

DMP Analysis Results and Visualization

Following DMP analysis, the results are visualized using a volcano plot to provide an overview of methylation differences between the selected conditions. Each point on the plot represents a CpG site, positioned according to its *deltaBeta* value on the x-axis and the negative logarithm of its *p*-value on the y-axis. This two-dimensional representation allows simultaneous evaluation of both the magnitude and statistical significance of methylation changes. CpG sites that exceed the user-defined Δβ and *p*-value thresholds are highlighted, with those exhibiting positive Δβ values classified as hypermethylated and those with negative Δβ values as hypomethylated. Dashed

reference lines mark the applied thresholds, clearly separating significant from non-significant CpG sites. The plot also labels the most prominent differentially methylated positions, allowing rapid identification of candidate loci for downstream biological interpretation. Through this visualization, users can intuitively assess how the chosen thresholds influence the overall distribution of methylation changes and identify CpG sites that contribute most strongly to the observed differences between the compared biological conditions. This visualization is implemented through the ggplot2 and ggrepel R packages.

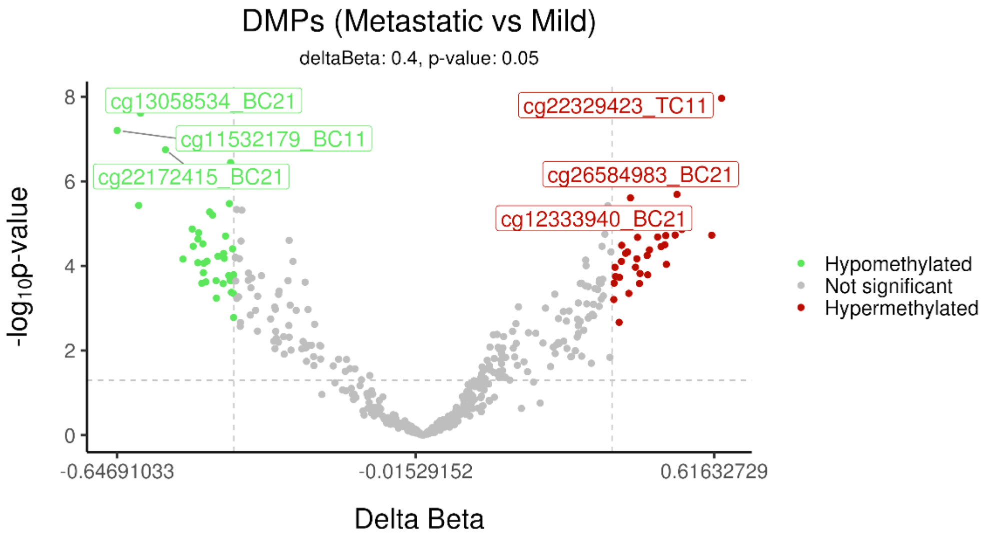

Supplementary Figure 6. Example of a volcano plot generated after differential methylation point (DMP) analysis using the *limma* package. In this example, the comparison is performed between the *Metastatic* and *Mild* categories, with user-defined thresholds of Δβ = 0.4 and *p*-value = 0.05. Each point represents a CpG site, plotted by its Δβ value on the x-axis and the negative logarithm of its *p*-value on the y-axis. Green points indicate hypomethylated sites, red points correspond to hypermethylated sites, and grey points represent CpG sites that do not meet the specified significance criteria. The labeled CpG sites denote the most statistically significant differentially methylated positions identified in this comparison.

Gene Mapping

After either DMP analysis or AI-based feature ranking, gene mapping is conducted to associate the selected CpG sites with their corresponding gene symbols. The process relies on Illumina methylation array annotations (450K, EPIC, or EPICv2) to accurately match each CpG identifier to its annotated gene. Based on the array type used, the CpG sites retained from the previous analytical step are cross-referenced with the appropriate annotation dataset, and the corresponding gene symbols are extracted. This step ensures that the selected CpG sites are linked to biologically meaningful entities, allowing the transition from probe-level findings to gene-level interpretation. The resulting output provides a comprehensive table containing each CpG site alongside its associated gene symbol, serving as the foundation for subsequent biological or functional analyses.

Enrichment Analysis and Visualization

Following gene mapping, enrichment analysis is performed using the Enrichr API to identify biological pathways and functional categories associated with the mapped genes. The implementation, developed in JavaScript, interfaces directly with Enrichr's cloud-based API to automate data upload, processing, and retrieval of enrichment results. Gene lists derived from the mapping step are read from CSV files, parsed through the PapaParse library, and uploaded to Enrichr using HTTP POST requests via the /addList endpoint. Once processed, enrichment results are fetched in tab-delimited format through the /export endpoint, enabling the extraction of key metrics such as *p-values*, *combined scores*, and *gene overlap counts*.

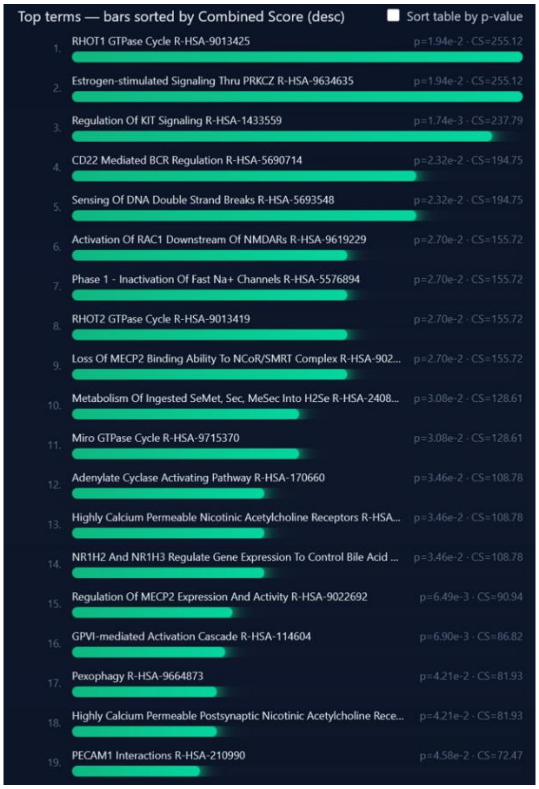

Supplementary Figure 7. Example of results of enrichment analysis after biomarker identification and gene mapping (as obtained within the application environment).

The tool dynamically retrieves available pathway libraries (e.g., Reactome, KEGG, WikiPathways, BioPlanet) through the Enrichr /datasetStatistics endpoint and allows users to select the preferred database for enrichment computation. The output is rendered interactively, displaying the top-ranked pathways based on statistical significance or combined score. This approach provides a reproducible and automated workflow that bridges methylation-based CpG selection with downstream functional interpretation, helping to identify biologically relevant pathways enriched in the selected gene sets.

Users should be aware of an important interpretive consideration when drawing conclusions from enrichment results. The enrichment analysis in CpGene operates at the level of gene symbols,

which are assigned to CpG sites based on the array manifest annotations. This means that a gene appearing in an enriched pathway was identified because one or more of its associated CpG sites ranked highly in the feature selection or DMP analysis step — not because the gene has been shown to be differentially expressed. The relationship between DNA methylation and transcriptional output is not uniform: hypermethylation at promoter CpG islands is generally linked to gene silencing, whereas methylation in gene bodies tends to correlate positively with expression, and the functional consequences of methylation at enhancers or other regulatory elements depend heavily on the specific genomic and cellular context. Furthermore, a single gene may be associated with multiple CpG sites showing divergent methylation patterns, so the direction of any regulatory effect cannot be inferred from enrichment results alone. Enriched pathways should therefore be treated as pointers to biological areas of interest rather than as direct evidence of pathway activation or suppression. Where matched gene expression data are available, integrating them with the methylation findings is strongly recommended before drawing functional conclusions.

Reproducibility and Translational Considerations

CpGene is designed as a discovery tool, and the CpG signatures it identifies should be regarded as candidates for further investigation rather than as validated biomarkers. Users intending to apply results in a translational or clinical context are encouraged to consider the following recommendations. The most important step toward confirming a finding is replication in an independent cohort. Results that hold across datasets collected at different sites, at different times, or using different sample batches are substantially more reliable than those observed in a single dataset. Where a second methylation array dataset is not available, targeted bisulfite sequencing of the top-ranked CpG sites offers an orthogonal method of quantifying methylation at those loci that is independent of the Illumina array platform and its associated technical characteristics. Cross-referencing identified sites with publicly available resources (such as the NCBI Gene Expression Omnibus, the Roadmap Epigenomics compendium, or ENCODE) can also help assess whether observed patterns are consistent with prior findings in related tissue types or disease contexts.

Within a single analysis, one practical way to increase confidence in a result is to run several of the ranking methods available in CpGene and focus on CpG sites that rank highly across multiple approaches. Because the methods differ substantially in their underlying assumptions and optimization objectives, consistent ranking across them is a stronger indicator of a genuine signal than ranking highly in a single method alone. For cross-platform validation, it is worth noting that while all supported array formats (450K, EPIC, EPICv2) share a large proportion of covered CpG sites, there are differences in probe sets and coverage. Users comparing results across platforms should restrict their analyses to CpG sites that are present and well-annotated on all platforms being compared.

Sample Size Considerations

DNA methylation array datasets typically contain orders of magnitude more features than samples, which presents a recurring challenge for both statistical testing and machine

learning–based analysis. While it is not possible to define universal minimum sample sizes (since the required sample size depends on the expected effect size, the degree of class imbalance, and the number of groups being compared) the following guidance reflects general experience with methylation array data and the properties of each implemented method. For DMP analysis using limma, the empirical Bayes variance shrinkage built into the framework is specifically designed to improve reliability when sample sizes are small, and it is possible to obtain meaningful results with as few as 3-4 samples per group when effect sizes are large and consistent. For more modest methylation differences, eight to ten samples per group is a more reasonable starting point, and power increases substantially beyond this threshold. Among the machine learning methods, Lasso and Ridge regression are generally the most tolerant of small sample sizes, since the regularisation penalty directly addresses the high-dimensionality problem by shrinking or eliminating coefficients. A minimum of around 10-15 samples per class is advisable to obtain stable estimates. The RFE-SVM shares a similar degree of robustness due to the margin-based formulation of the SVM, and comparable sample sizes apply. Random Forest and SHAP XGBoost tend to require somewhat more data in the range of 15-20 samples per class to build stable ensembles without individual trees overfitting to noise. The Garson-Olden MLP is the most sensitive to sample size among the implemented methods, owing to the larger number of trainable parameters relative to the others; at least twenty to twenty-five samples per class is recommended, and the use of early stopping and seed-averaging (both enabled by default) helps to reduce instability when data are limited.

Additional Material

A detailed video/showcase of how the application works with example data has been created and uploaded on youtube. Currently the video is only accessible to select audiences that have a link to it. The video can be found here.

References

Garson, G. D. (1991). *Interpreting neural network connection weights.* Artificial Intelligence Expert, 6(4), 46–51.

Olden, J. D., Jackson, D. A., & Joy, M. K. (2004). *An accurate comparison of methods for quantifying variable importance in artificial neural networks using simulated data.* Ecological Modelling, 178(3–4), 389–397.

Azman NS, Samah AA, Lin JT, Majid HA, Shah ZA, Wen NH, Howe CW. Support vector machine–Recursive feature elimination for feature selection on multi-omics lung cancer data. Progress In Microbes & Molecular Biology. 2023 Apr 4;6(1).

Huang Z, Chen D. A breast cancer diagnosis method based on VIM feature selection and hierarchical clustering random forest algorithm. IEEE Access. 2021 Dec 30;10:3284-93.

Khan MH, Bhadra A, Howlader T. Stability selection for lasso, ridge and elastic net implemented with AFT models. Statistical applications in genetics and molecular biology. 2019 Oct 1;18(5).

Zhang J, Ma X, Zhang J, Sun D, Zhou X, Mi C, Wen H. Insights into geospatial heterogeneity of landslide susceptibility based on the SHAP-XGBoost model. Journal of environmental management. 2023 Apr 15;332:117357.

Friedman JH, Hastie T, Tibshirani R. Regularization paths for generalized linear models via coordinate descent. Journal of statistical software. 2010 Feb 2;33:1-22.

Greenacre M, Groenen PJ, Hastie T, d'Enza AI, Markos A, Tuzhilina E. Principal component analysis. Nature Reviews Methods Primers. 2022 Dec 22;2(1):100.

Adams C, Nair N, Plant D, Verstappen SM, Quach HL, Quach DL, Carvidi A, Nititham J, Nakamura M, Graf J, Barton A. Identification of Cell-Specific Differential DNA Methylation Associated With Methotrexate Treatment Response in Rheumatoid Arthritis. Arthritis & rheumatology. 2023 Jul;75(7):1088-97.

Nissenbaum, J., Bar-Nur, O., Ben-David, E., & Benvenisty, N. (2013). Global indiscriminate methylation in cell-specific gene promoters following reprogramming into human induced pluripotent stem cells. *Stem Cell Reports*, *1*(6), 509-517.
